# Early-life fecal microbiome and metabolome dynamics in response to an intervention with infant formula containing specific prebiotics and postbiotics

**DOI:** 10.1152/ajpgi.00079.2021

**Published:** 2022-03-29

**Authors:** Alfonso Rodriguez-Herrera, Sebastian Tims, Jan Polman, Rocío Porcel Rubio, Antonio Muñoz Hoyos, Massimo Agosti, Gianluca Lista, Luigi T. Corvaglia, Jan Knol, Guus Roeselers, Juan L. Pérez Navero

**Affiliations:** ^1^Instituto Hispalense de Pediatria, Sevilla, Spain; ^2^Danone Nutricia Research, Utrecht, The Netherlands; ^3^Servicio de Pediatría, Hospital Quiron, Barcelona, Spain; ^4^Department of Pediatrics, Hospital Clínico Universitario San Cecilio, Granada, Spain; ^5^Neonatologia e Terapia Intensiva Neonatale, Polo Universitario F. Del Ponte, Varese, Italy; ^6^Terapia Intensiva Neonatale, Ospedale dei Bambini Vittore Buzzi, ASST-FBF-Sacco, Milano, Italy; ^7^Intensive Therapy Unit, Hospital S. Orsola Malpighi, Bologna, Italy; ^8^Department of Microbiology, Wageningen University, Wageningen, The Netherlands; ^9^Pediatrics Department, Reina Sofia University Hospital, Maimonides Institute for Biomedical Research, CIBERER, Cordoba, Spain

**Keywords:** early life, gut microbiota, infant formula, metabolomics

## Abstract

This study examined fecal metabolome dynamics to gain greater functional insights into the interactions between nutrition and the activity of the developing gut microbiota in healthy term-born infants. The fecal samples used here originate from a randomized, controlled, double-blind clinical study that assessed the efficacy of infant formula with prebiotics and postbiotics (experimental arm) compared with a standard infant formula (control arm). A group of exclusively breast-fed term infants was used as a reference arm. First, conventional targeted physiological and microbial measurements were performed, which showed differences in fecal *Bifidobacterium* levels and corresponding activity (e.g., lactate levels). Next, the overall fecal microbiota composition was determined by 16S rRNA gene amplicon sequencing. The microbiota composition profiles showed several bacterial groups in the experimental arm to be significantly different from the control arm and mostly closer to the levels observed in the reference arm. Finally, we applied an untargeted UPLC-MS/MS approach to examine changes in the fecal metabolome. Fecal metabolome profiles showed the most distinct separation, up to 404 significantly different metabolites, between the study arms. Our data reveal that infant formula with specific prebiotics and postbiotics may trigger responses in the intestinal microbiota composition that brings the ensuing fecal metabolite profile of formula-fed infants closer toward those observed in breast-fed infants. Furthermore, our results demonstrate a clear need for establishing an infant gut metabolome reference database to translate these metabolite profile dynamics into functional and physiologically relevant responses.

**NEW & NOTEWORTHY** Untargeted metabolomics techniques can provide a “snapshot” of an ecosystem in response to environmental stimuli, such as nutritional interventions. Our analyses of fecal samples from infants demonstrate the potential of phenotyping by metabolomics while deciphering the complex interactions of early-life nutrition and gut microbiome development.

## Graphical abstract

**Figure:**
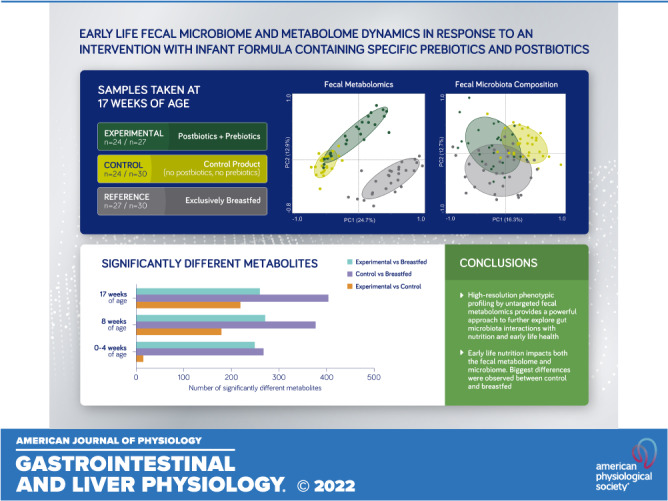

## INTRODUCTION

The human gut harbors a complex microbial ecosystem, the gut microbiota, that has been recognized as an essential part of our human physiology (1–6). In human adults, the microbiota is considered to be a relatively stable ecosystem (7, 8). The microbial colonization process in early life is heavily intertwined with the maturation of the gastrointestinal tract itself. Therefore early-life colonization can be considered a fundamental step in healthy development (9). Several early-life environmental factors have been shown to have a pervasive and long-lasting impact on the gut microbiota composition and activity (10), thereby increasing the risk of diseases in later life (11, 12). Early-life nutrition is a major factor that impacts the developing gut microbiota community. *Bifidobacterium* species typically dominate the gut microbiota of breast-fed infants, whereas a microbiota that is richer in members of the phylum Firmicutes (Bacillota) is typically observed in infants fed with generic cow’s milk-based formula without prebiotics (13).

In this age of high-throughput omics-based technologies, increasingly sophisticated tools have become available to study the gut microbiota at different molecular levels. In the past decade, the most widely used tools to investigate the gut microbiota have been based on metagenome sequencing or on sequencing of amplicons of bacterial 16S rRNA genes to identify which taxonomic lineages are present.

In recent years, high-throughput biological technologies (also known as “omics”) have begun to revolutionize many fields of biomedicine. Among the most widely used are genomics and transcriptomics, measuring DNA sequences and gene expression, respectively. A limitation of microbiota profiling by DNA-based methodologies is that it only provides a potential functional capacity; however, it does not provide a direct view of the actual metabolic output of the gut ecosystem.

Metabolomics, a relative newcomer to the “omics” field, is an omics approach where easily hundreds of metabolites (usually small molecules <1,000–1,500 Da) are measured simultaneously in biological samples with the goal of identifying metabolic pathways that are activated or deactivated in health or disease. As such, it fills an important gap in understanding the functions of genes and proteins. Hence, this form of high-resolution phenotypic profiling is now pushing this field of research beyond “description” and into “function and mechanism” (14, 15). After all, metabolite profiles portray a functional phenotype that results from the culmination of all activated genes, their (epigenetic) expression modifications and other transcriptional regulations, their subsequent posttranslational protein modifications, and the (local) environmental factors.

The current consensus in the gut microbiome research field is that nutrition and gut microbiome interactions affect host health via microbiota-host cometabolic networks. As such, different nutritional regimes are expected to cause divergent metabolomes, which reflect different prioritization of functional pathways among the microbial communities. Hence, metabolomics may provide an integrative understanding of nutrition-host-microbiota interactions, as it allows us to characterize the key biochemical changes caused by nutritional interventions. In this study, we investigated the relevance of fecal metabolomics by comparing conventional targeted physiological measurements, targeted microbiota quantification, and 16S rRNA gene amplicon sequencing with the information obtained from untargeted fecal metabolite profiling on the fecal samples collected within a clinical trial that compared an infant formula with specific prebiotics and postbiotics with a control formula. Postbiotics being defined here as bioactive compounds produced by food-grade microorganisms in a fermentation process, based on Aguilar-Toalá and coworkers (16, 17). These bioactive compounds support the development of the early-life gut microbiota and immune system. The aim of this study was to examine fecal metabolome dynamics in response to a clinical intervention to gain greater functional insights into the interactions between nutrition, specifically prebiotics and postbiotics used, and the activity of the developing gut microbiota in young infants.

## MATERIALS AND METHODS

### Samples

The samples used here originate from a randomized, controlled, double-blind study (Netherlands Trial Register: NTR3455; the LIFE study) that was previously published (18). This trial tested the efficacy of an infant milk formula with a prebiotic mix containing 90% short-chain galactooligosaccharides and 10% long-chain fructooligosaccharides (scGOS/lcFOS; 0.8 g/100 mL, 9:1) and postbiotics derived from the Lactofidus fermentation process (18). Postbiotics being defined here as bioactive compounds produced by food-grade microorganisms in a fermentation process including microbial cells, cell constituents, and metabolites, which support health and/or well-being, based on Aguilar-Toalá and coworkers (16). In the experimental formula, the postbiotics were generated by subjecting 30% of the total formula composition to a unique fermentation process (Lactofidus) involving two bacterial strains, *Bifidobacterium breve* C50 and *Streptococcus thermophilus* 065. One of the bioactive compounds that was generated in this process is 3′-galactosyllactoses (3′-GL), an oligosaccharide found in human milk, at a final level of ∼25 mg/100 mL formula. The efficacy of the formula containing specific prebiotics and postbiotics was compared with that of a nonfermented infant formula without prebiotics and postbiotics (control) (18). The compositions of these formulas were isocaloric and follow Directive 2006/141/EC (see Table 1). Furthermore, this trial consisted of a group of exclusively breast-fed term infants as a reference arm. This study was conducted according to the International Conference on Harmonization Good Clinical Practice (ICH-GCP) principles and in full compliance with the principles of the Declaration of Helsinki (59th WMA General Assembly, Seoul, October 2008) and with the local laws and regulations of the country where the study was performed.

**Table 1.** Product composition of the infant formulas used from the randomized, controlled, double-blind study (Netherlands Trial Register: NTR3455)

	Experimental	Control
Energy (kcal/100 mL formula)	67	67
Carbohydrates (g/100 mL formula)	7.5	7.8
Glucose	0.3	0
Lactose	7.1	7.6
Prebiotics (g/100 mL formula)	0.8	0
Fiber (g/100 mL formula)	0.6	0
Postbiotics (% of dry weight)	30	0

Samples were collected at randomization or the day thereafter (0–4 wk of age; baseline), at 8 wk of age, and at 17 wk of age no later than 1 day after the last intake of the study product. There was no use of concomitant drinks or foods before 17 wk of age in the study population. Caregivers were instructed to collect a fecal sample in the week before a visit to the study site. Samples were collected from the diaper in a 10-mL sterile collection tube (no preservatives) at home and were frozen at −12°C or colder immediately. After the samples were given to the investigational staff at the study site, the samples were stored at −18°C or, if possible, at −80°C. Complete sample sets were shipped on dry ice to the laboratory of Danone Nutricia Research (Utrecht, The Netherlands) and stored at −80°C for later analysis.

The fecal parameters were analyzed in a subgroup of infants who were selected based on the following criteria: natural birth (vaginal delivery); no use of probiotics, milk thickeners, antibiotics, or other medication that could influence the microbiota development from birth until the end of the study participation; and no use of laxatives 3 days or less before fecal sampling. This subgroup consisted of 90 subjects (30 from each of the three study arms) for a total of 264 stool samples. Subject demographics and baseline characteristics that could affect the gut microbiota, such as gestational age, birth weight, mother’s age, ethnicity, and the number of siblings in the household, are listed in Supplemental Table S1 (https://doi.org/10.6084/m9.figshare.14729682.v1) and were balanced over the study groups. As not all samples contained enough fecal mass to perform all analysis, a breakdown of the number of samples measured per study arm, per time point, and per analysis type is provided in Table 2. Once all samples were available, they were thawed once and aliquoted for the various different types of analyses.

**Table 2.** Number of fecal infant samples measured per analysis type

Time Point	Experimental	Control	Breast-Fed
16S rRNA gene amplicon sequencing			
0–4 wk of age	30	30	30
8 wk of age	28	30	29
17 wk of age	27	30	30
Physiological and targeted microbiota data			
0–4 wk of age	28	29	28
8 wk of age	28	30	28
17 wk of age	24	29	29
Metabolomic			
0–4 wk of age	20	25	25
8 wk of age	17	17	19
17 wk of age	24	24	27

### Targeted Physiological and Microbial Data

In the selected set of fecal samples, the following targeted physiological and microbial parameters were measured: pH, short-chain fatty acid (SCFA) levels (i.e., acetate, propionate, butyrate, isobutyrate, valerate, and isovalerate), d- and l-lactate, secretory immunoglobulin A (sIgA), calprotectin, and presence of *Clostridioides difficile*. The quantification methodology of these parameters has been described in more detail previously (19).

### DNA Extraction

DNA extraction from stool samples was performed with the QIAmp DNA Stool Mini Kit (Qiagen) according to the manufacturer’s protocol except for the addition of two bead-beating steps. To 0.2–0.3 g of fecal sample, 300 mg of 0.1-mm glass beads was added together with 1.4 mL of ASL (lysis) buffer, and on this suspension, the first bead-beating step was applied thrice for 30 s (FastPrep-24 instrument program 5.5). After the addition of the InhibitEx tablet, the second bead-beating step was applied thrice for 30 s (FastPrep-24 instrument program 5.5) to homogenize the sample. After each bead-beating step, samples were cooled for 5 min on ice. Extracted DNA purity was checked using the NanoDrop spectrophotometer (Thermo-Fisher Scientific, Inc.), whereas DNA quality and concentration were measured using the Quant-iTTM 193 dsDNA BR Assay kit (Invitrogen). DNA aliquots were stored at −80°C until use.

### Amplicon Sequencing Analysis

From the purified fecal DNA extracts, the V3–V5 regions of the bacterial 16S rRNA gene were amplified, using primers 357 F and 926Rb. A 454 FLX Sequencer (454 Life Sciences, Branford, CT) was used to sequence the obtained 16S rRNA gene amplicons, as described previously (20).

The Quantitative Insights Into Microbial Ecology (QIIME) pipeline version 1.8.0 was used to analyze the sequence data (21). Quality control filters were set to discard sequences with a length below 200 bases, with a length above 1,000 bases, with a mean sequence quality score of less than 25, with any ambiguous bases, or containing homopolymer stretches of more than six bases. Chimeric sequences were filtered with QIIME’s own ChimeraSlayer. On the filtered sequences, de novo Operational Taxonomic Unit (OTU) picking was applied using the USEARCH algorithm (22), which grouped sequences with ≥97% identity. Subsequently, the Ribosomal Database Project Classifier (RDP) (23) was applied to assign taxonomy to the representative sequences (i.e., the most abundant sequence) of each OTU by alignment to the SILVA ribosomal RNA database (release version 1.0.8) (24).

### Metabolomic Profiling

Frozen fecal aliquots were shipped under dry ice to a commercial laboratory (Metabolon, Durham, NC) for metabolite analysis. Procedures for metabolic profiling have been described previously (25) for the three platforms used in combination for the analysis, including GC-MS, polar LC, and two LC-MS/MS systems, one optimized for positive ionization and one optimized for negative ionization. See Supplemental Table S2 for the detected metabolites and the platform from which their data were extracted. Proprietary software was used to match ions to an in-house library of standards (Metabolon, Durham, NC) for metabolite identification and for metabolite quantitation by using the area-under-the-curve approach. A number of internal standards were added, and platform variability was determined by calculating the median relative standard deviation (RSD) for these internal standards, which was 5%. Because these standards are added to the samples immediately before injection into the instrument, this value reflects instrument variation. In addition, the median RSD for the metabolites that were consistently measured in a pool, created from small aliquots of each measured sample, was 9%. Data were collected over multiple platform-run-days and were adjusted by scaling to the median values for each group-balanced run-day block for each individual compound (see Supplemental Table S3). This approach minimizes any interday instrument gain or drift but does not interfere with intraday sample variability. Data were not otherwise adjusted or normalized. The normalized peak area counts were rescaled for each detected metabolite to have a median equal to 1, and subsequently, missing values were imputed with the minimum value.

### Statistics

For the targeted physiological and microbial parameters, the values below the quantification limit were replaced by (detection limit + quantification limit)/2, whereas the values below the detection limit were replaced by detection limit/square root of 2. If the percentage of values of a given parameter were detected in 70% or more of the samples, then the parameter was treated as continuous data; otherwise, the parameter was converted to binary (1 indicating presence, 0 indicating absence, and/or below detection limit). For all continuous parameters, a Wilcoxon rank-sum test was used to calculate *P* values for the difference between experimental and control at each time point. For all binary parameters, the χ^2^ test (Fisher’s exact if expected cell counts <5) was used for inference making.

For the 16S rRNA gene amplicon sequencing results, the relative abundances of each taxon at genus level subjected to one of the following tests, depending on the distribution of zero and nonzero values across the study arms, were performed: a Wilcoxon rank-sum test was performed when only nonzero counts were observed or when there was at least one expected zero count <5 and, at the same time, at least one expected nonzero count is <5; a two-part statistics test (26) was performed if both groups have ≥10 nonzero values; if either group has <10 nonzero values, the data are treated as binary and a χ^2^ test was performed unless 50% of the cells has expected counts <5; in which case, a Barnard test was performed.

For the metabolomics results, the normalized and rescaled signals were subjected to the two-part statistics test (26).

Both the statistical testing on 16S rRNA gene amplicon sequencing data and that on the metabolomics data resulted, for comparison, in a large set of *P* values and, therefore, were corrected for multiple testing by assessing the positive false discovery rate (pFDR) (27). The bootstrap method described by Storey et al. (28) is used to estimate π_0_ and subsequently calculate *q* values, a measure of each feature’s significance. Results of both the 16S rRNA amplicon sequencing and the metabolomics were considered to be statistically significant when both the *P* value and the *q* value were <0.05.

Principal coordinate analysis (PCoA), using Bray–Curtis metrics, was performed on both the 16S rRNA gene amplicon sequencing data and the metabolomics data using the Canoco (version 5.10) software for multivariate data exploration (29).

## RESULTS

To assess the contribution of metabolomics data to the integrative understanding of nutrition-host-microbiota interactions in infants, this study compared conventional targeted physiological measurements, targeted microbiota quantification, and 16S rRNA gene amplicon sequencing with the information obtained from untargeted fecal metabolite profiling. Samples used here originate from a randomized, controlled, double-blind nutritional intervention study (30). In this clinical trial, the efficacy of a formula with specific prebiotics and postbiotics against a control formula was determined. In addition, this trial included a group of exclusively breast-fed term infants as a reference arm. For this trial, the primary outcome parameters of growth and safety have previously been described (30).

### Physiological and Targeted Microbiota Data Confirm Prebiotic Effect

**Figure 1.**
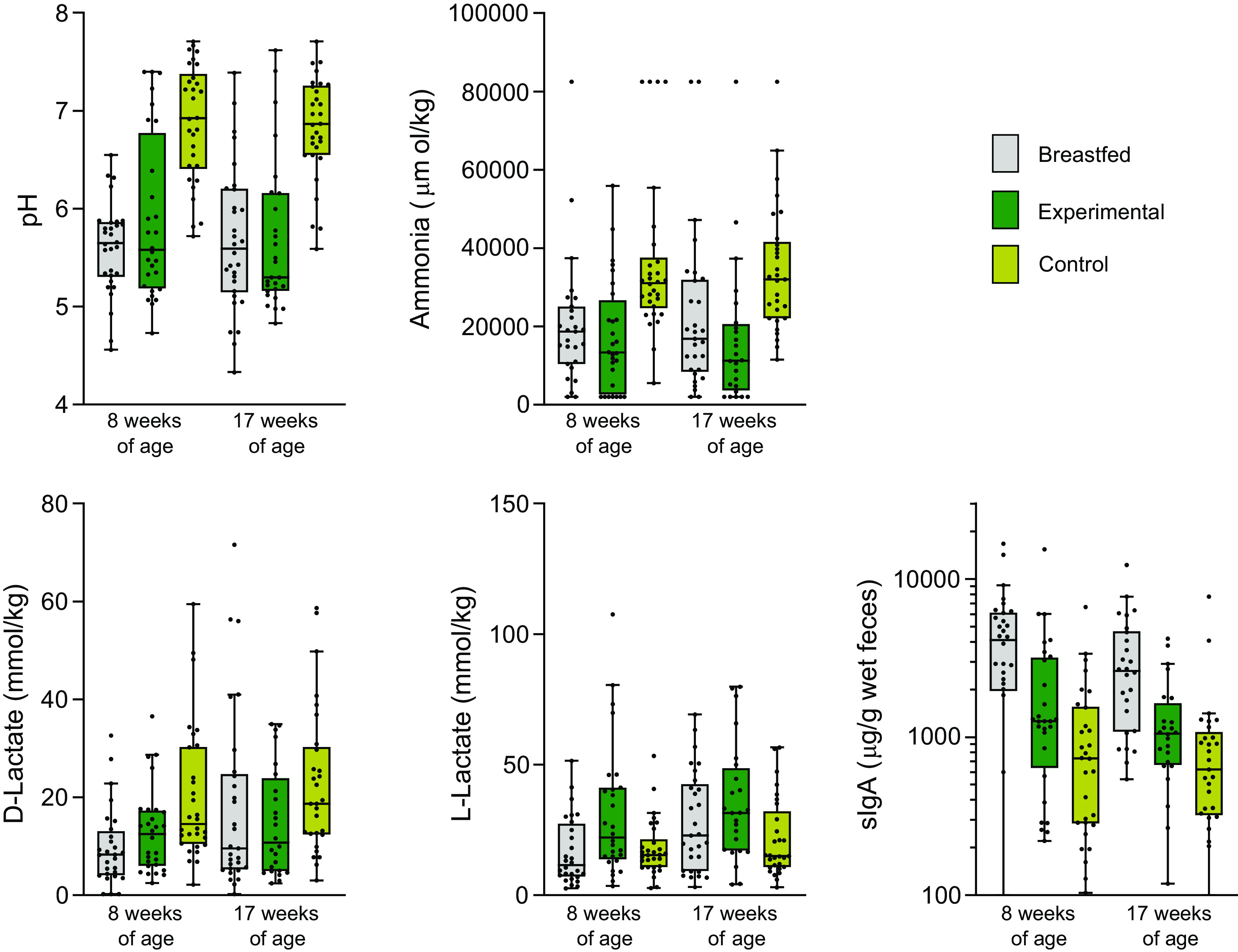
Detected levels of pH, ammonia, d-lactate, l-lactate, and sIgA in feces from healthy term*-*born infants at 8 wk and 17 wk of age. Gray, breast-fed (*n* = 28 at 8 wk of age, 16/12 male/female; *n* = 29 at 17 wk of age, 17/12 male/female); green, experimental (*n* = 28 at 8 wk of age, 16/12 male/female; *n* = 24 at 17 wk of age, 12/12 male/female); and yellow, control (*n* = 30 at 8 wk of age, 16/14 male/female; *n* = 29 at 17 wk of age, 15/14 male/female). sIgA, secretory immunoglobulin A.

The use of this specific prebiotics and postbiotics mixture in infant milk formulas has previously been reported to modulate the gut microbiota with at least the effects known to occur after intake of the prebiotics by infants (19). Here, targeted physiological measurements confirmed these effects, that is, compared with the control group, the fecal samples from the experimental group at 17 wk of age showed a lower pH, lower amounts of ammonia, higher amounts of acetate and l-lactate, lower amounts of propionate and d-lactate, lower occurrence of (iso-)butyric acid and (iso-)valeric acid, and higher levels of sIgA (Fig. 1; Supplemental Fig. S1; Supplemental Table S6). None of these parameters was different at baseline (Supplemental Table S4). Furthermore, the targeted microbiota quantification by qPCR was in line with these results, that is, compared with the control arm, the samples from the experimental arm at 17 wk of age showed an increase in members of the genus *Bifidobacterium* and a lower occurrence of members of the *Clostridioides difficile* group (Supplemental Fig. S1; Supplemental Table S6). Moreover, these measurements already showed significant differences during the trial when the infants were 8 wk of age, and therefore 4–8 wk on the formulas, except for the presence of *Clostridioides difficile* (Supplemental Fig. S1; Supplemental Table S5).

### Experimental Formula Moves Microbiota Composition toward That of Breast-Fed Infants

**Figure 2.**
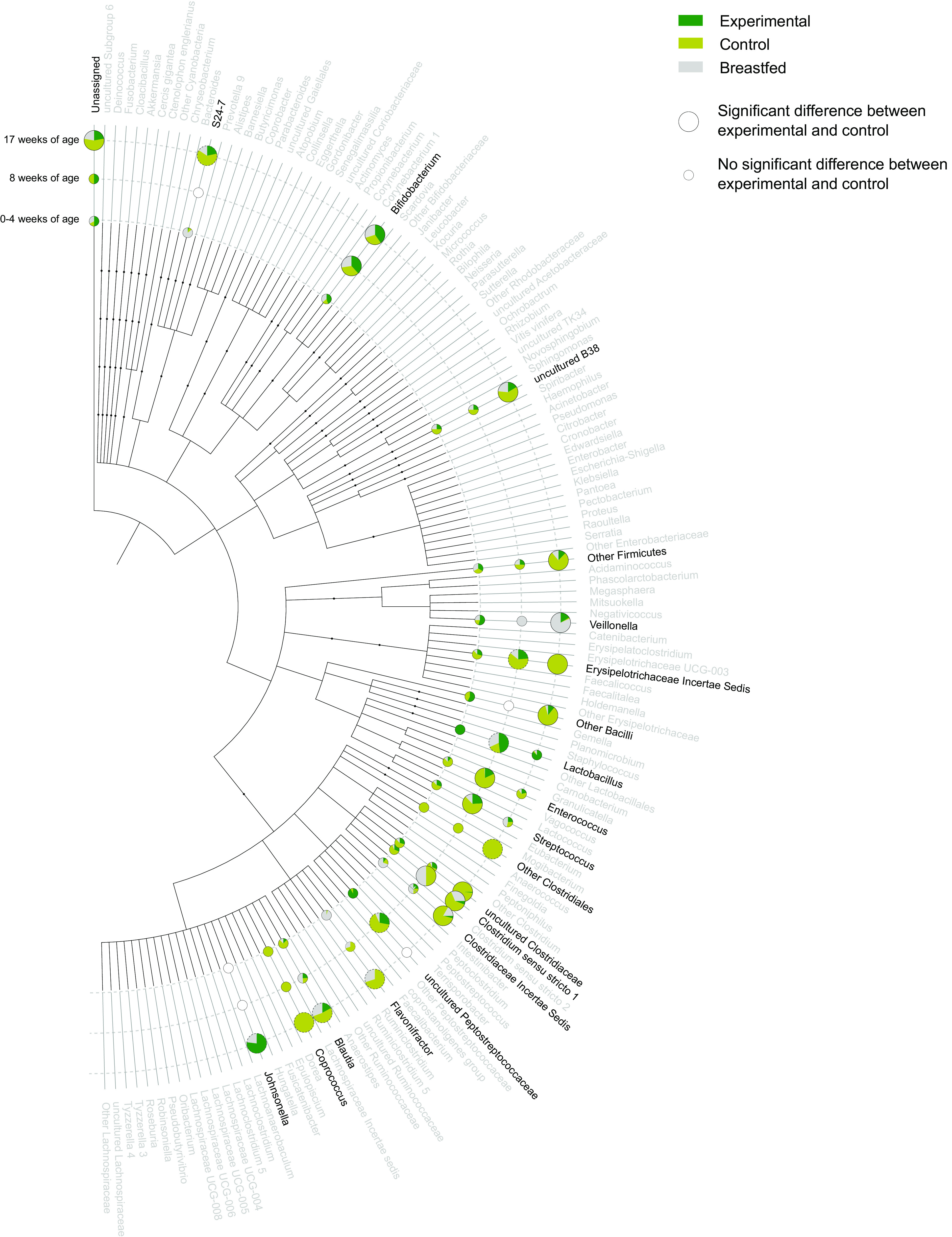
Dendrogram showing the taxonomic relation of all microbial genera detected by 16S rRNA gene sequencing in infant fecal samples. Infants consumed an infant formula with prebiotics and postbiotics (experimental) or a standard infant formula without prebiotics or postbiotics (control) or were breast-fed. Genera were visualized with pie charts when there was a significant difference at any of the time points, that is, at 0–4 wk of age (baseline sample), 8 wk of age, or at 17 wk of age. The pie charts visualize either the median abundance value for each study arm (when both experimental and control have <10 zero values) or the prevalence for each study arm (when both experimental and control have ≥10 zero values). Pie charts reflecting the prevalence have a gray dashed outer border. Green, experimental; yellow, control; and gray, breast-fed. Empty pie charts indicate a time point in which the genus was not detected in any of the samples.

**Figure 3.**
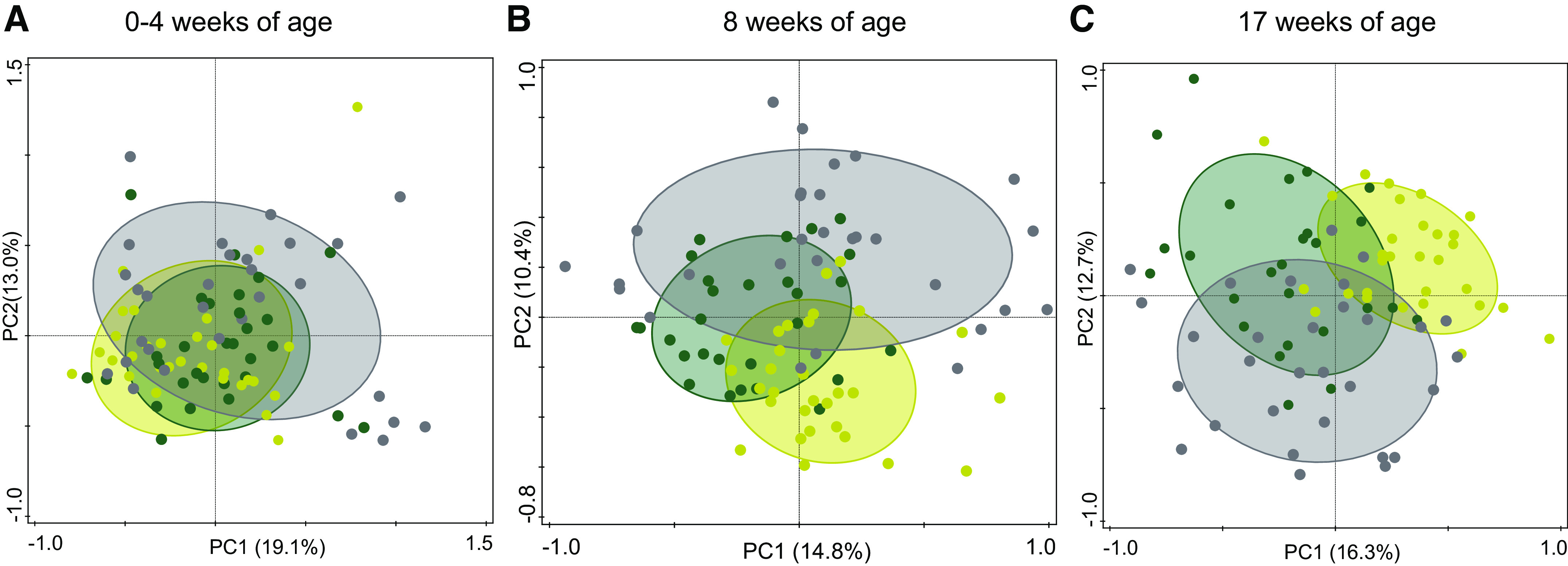
PCoA plots 16S rRNA gene pyrosequencing data. PCoA (Bray*–*Curtis) on the 16S rRNA gene pyrosequencing data at the genus level, separately performed for the signals of the three time points: at 0–4 wk of age (baseline; *left*), at 8 wk of age (*middle*), and at 17 wk of age (*right*). Gray, breast-fed (*n* = 29 at 8 wk of age, 16/13 male/female; *n* = 30 at 17 wk of age, 17/13 male/female); green, experimental (*n* = 28 at 8 wk of age, 16/12 male/female; *n* = 27 at 17 wk of age, 15/12 male/female); and yellow, control (*n* = 30 at 8 wk of age, 16/14 male/female; *n* = 30 at 17 wk of age, 16/14 male/female). PCoA, principal coordinate analysis.

Untargeted 16S rRNA gene amplicon sequencing revealed several bacterial taxa (7 genera at 8 wk of age, 16 genera at 17 wk of age) that had a significantly different abundance in infants on experimental formula compared with those on control formula. These differences were in line with the microbial community composition and activity changes indicated by the physiological measurements and qPCR analyses. Especially in the 17 wk of age samples, the majority of these significantly different bacterial groups appear to be more in line with the levels detected in the breast-fed reference arm, such as *Bacteroides* S24_7, uncultured gamma proteobacterium B38, *Blautia*, and several uncultured taxa from the Firmicutes phylum (Fig. 2; Supplemental Table S7). Principal coordinate analysis (PCoA) of the 16S data (Fig. 3) revealed no clear separation between the formula-fed infants and the breast-fed infants at baseline. However, compared with the situation at baseline, the microbiota composition showed more separation for all three arms during (at 8 wk of age) and after (at 17 wk of age) the trial.

### Metabolomics Signatures Distinctively Separate Intervention Arms

**Figure 4.**
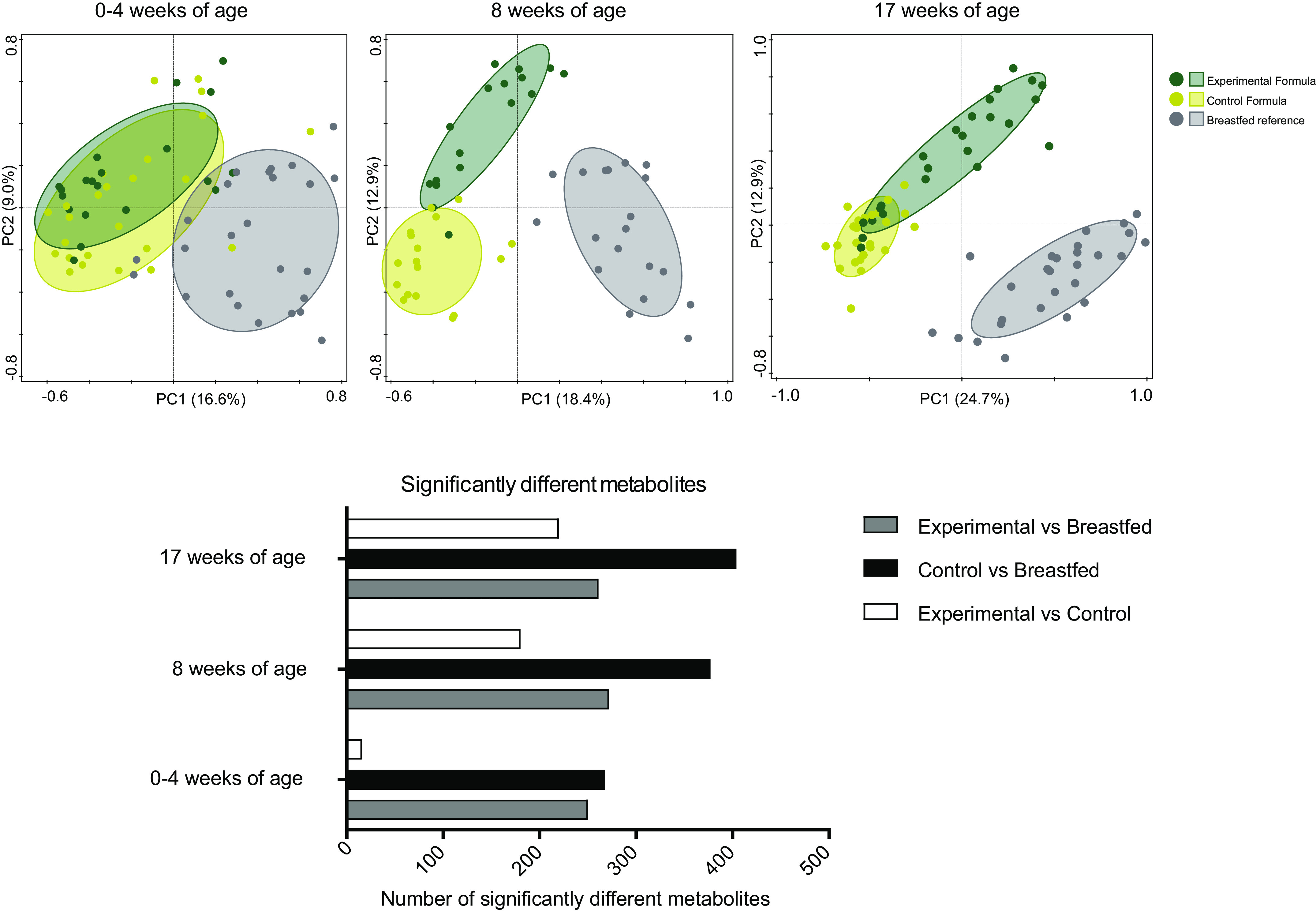
*Top*: PCoA (Bray*–*Curtis) on the standardized and normalized metabolite signals, separately performed for the signals of each time point, that is, at 0–4 wk of age (baseline; *top left*), at 8 wk of age (*top middle*), and at 17 wk of age (*top right*). Gray, breast-fed (*n* = 19 at 8 wk of age, 11/8 male/female; *n* = 27 at 17 wk of age, 15/12 male/female); green, experimental (*n* = 17 at 8 wk of age, 10/7 male/female; *n* = 24 at 17 wk of age, 15/9 male/female); and yellow, control (*n* = 17 at 8 wk of age, 8/9 male/female; *n* = 24 at 8 wk of age, 11/13 male/female). *Bottom*: number of significantly different metabolites between each pair of feeding groups is visualized as bar plots, separated per time point. PCoA, principal coordinate analysis.

Unbiased gut metabolomic profiling by UPLC-MS/MS was applied to profile the metabolic changes in feces between the different intervention groups. A total of 786 metabolites (470–625 unique metabolites per sample) were identified. PCoA of the metabolome data revealed a clear separation between the formula-fed infants and the breast-fed infants at baseline, reflecting an expected nutritional intake due to feeding mode at baseline (Fig. 4). However, a clear separation between all three arms occurred during and after the trial (Fig. 4). The observed separations in the metabolomics PCoA plots cover more variation within the data, that is, the first two principal components account for more variability, than the separation observed by PCoA analysis of the 16S data (Fig. 3).

The identified metabolites showed significant differences in abundance already at baseline between the breast-fed infants and either the control group (268 metabolites) or the experimental group (250 metabolites). Not many metabolites showed significant differences between the two intervention arms at baseline (i.e., only 16 metabolites were significantly different; Fig. 4). Interestingly, although not immediately evident from the PCoA plots (Fig. 4), the number of significantly different metabolites increased in time in the control arm up to 404 metabolites, whereas the number of differential metabolites between the experimental and breast-fed reference arm remains more or less constant throughout the trial, that is, 261 metabolites at study end (Fig. 4). This suggests that besides a baseline difference between (any) formula feeding and breastfeeding, the consumption of the control formula continuously drives the microbiota functionality away from that in human milk-fed infants. In contrast, although the fecal microbiota functioning in infants receiving experimental formula with specific prebiotics and postbiotics is not identical to that of the fecal microbiota of breast-fed infants, the observed baseline differences did not increase in the 3- to 4-mo time period investigated here.

## DISCUSSION

The relevance of unbiased metabolomics on fecal samples was shown and compared with conventional physiological measurements and sequence-based taxonomic profiling. To this end, a subset of fecal samples was used from a randomized, controlled, double-blind study that assessed the efficacy of an infant formula with a specific prebiotic, scGOS/lcFOS mixture, and postbiotics. Formulas supplemented with this prebiotic scGOS/lcFOS mixture have been shown to modulate the gut microbiota composition toward a *Bifidobacterium* spp rich community and improve immune functionality (31–33). The effects of this scGOS/lcFOS mixture on the gut microbiota were previously shown to be maintained when combined with these specific postbiotics (19), and this combination was shown to promote an improved clinically relevant reduction of infantile colic (34). Here, using conventional physiological measurements and targeted microbiota quantification, we confirm the modulation of the gut microbiota properties, such as lowering the pH, modifying the organic acid profile, and increasing the *Bifidobacterium* spp counts. A comparison of 16S rRNA amplicon profiles of the two intervention arms revealed more detailed changes in the microbiota composition. Most of the bacterial genera that were differentially abundant between the experimental and control groups at 17 wk of age showed that the median abundances in the experimental arm with prebiotics and postbiotics were closer to those observed in the breast-fed reference arm. These changes appear to manifest at a slower pace compared with the changes observed using conventional targeted physiological parameters, most of which are already significantly different at 8 wk of age. The ecological mechanisms driving these differences are difficult to deduce with current knowledge, although many taxa that were reduced in the experimental arm are known to be more characteristic of an adult microbiota and are mostly absent, or at least reduced, in the microbiota of breast-fed infants. Indeed, these taxa showed a lower abundance in the breast-fed reference group. Interestingly, three of these taxa—the *Blautia* genus, an uncultured taxon within the order Clostridiales, and an uncultured taxon within the order Erysipelotrichales—were also found to be reduced in the fecal samples from infants consuming a similar infant formula with the same prebiotics and postbiotics from a previous trial (35). These were reduced only with the combination of the prebiotics and postbiotics and not with either prebiotics or postbiotics alone (35). Previous findings have indicated that members of the genus *Blautia* are capable of metabolizing hydrogen (H_2_) and CO_2_ to acetate (36, 37). H_2_ is a general by-product of colonic fermentation. Recent findings have highlighted the importance of a proper equilibrium between H_2_-producing and H_2_-utilizing bacteria, where an imbalance between these two bacterial functions seems to be associated with discomfort for the host (38). Although H_2_ is only suspected to have neuromediator functionality (39), it could be used for H_2_S formation, which is known for its neuromediator functionality (39, 40). Therefore, the *Blautia* levels presented here could be a marker for the H_2_ cycle of the gut and may be indicative of a proper microbial metabolism.

From the total number of genera, relatively few responded consistently to the type of formulas received (Fig. 2). In contrast, the metabolomics data revealed many more parameters with clear distinguishable signals between the two formulas. From 23.7% up to 51.9% of the detected metabolites were different between the formula arms and/or the breast-fed reference at any time point (Fig. 4), except for the baseline differences between experimental and control, where only 16 metabolites (i.e., 2.1% of the detected metabolites) were significantly different. Such a low number of differential metabolites was expected, as the corresponding samples were taken around the time that the subjects were randomized over the two intervention arms. Hence, the metabolomics data are in line with the targeted physiological data and confirm that the microbiome functionality is more dynamic and responsive to nutrition compared with taxonomic microbiota composition. Similar findings have been reported in a study by Bazanella and colleagues (41), which showed a more distinct separation in the fecal metabolite profiles between formula-fed and human milk-fed infants in early life. This difference in dynamics and responsiveness might not come as a surprise, as it takes some time before changes in microbial activity lead to changes in microbial cell numbers.

**Figure 5.**
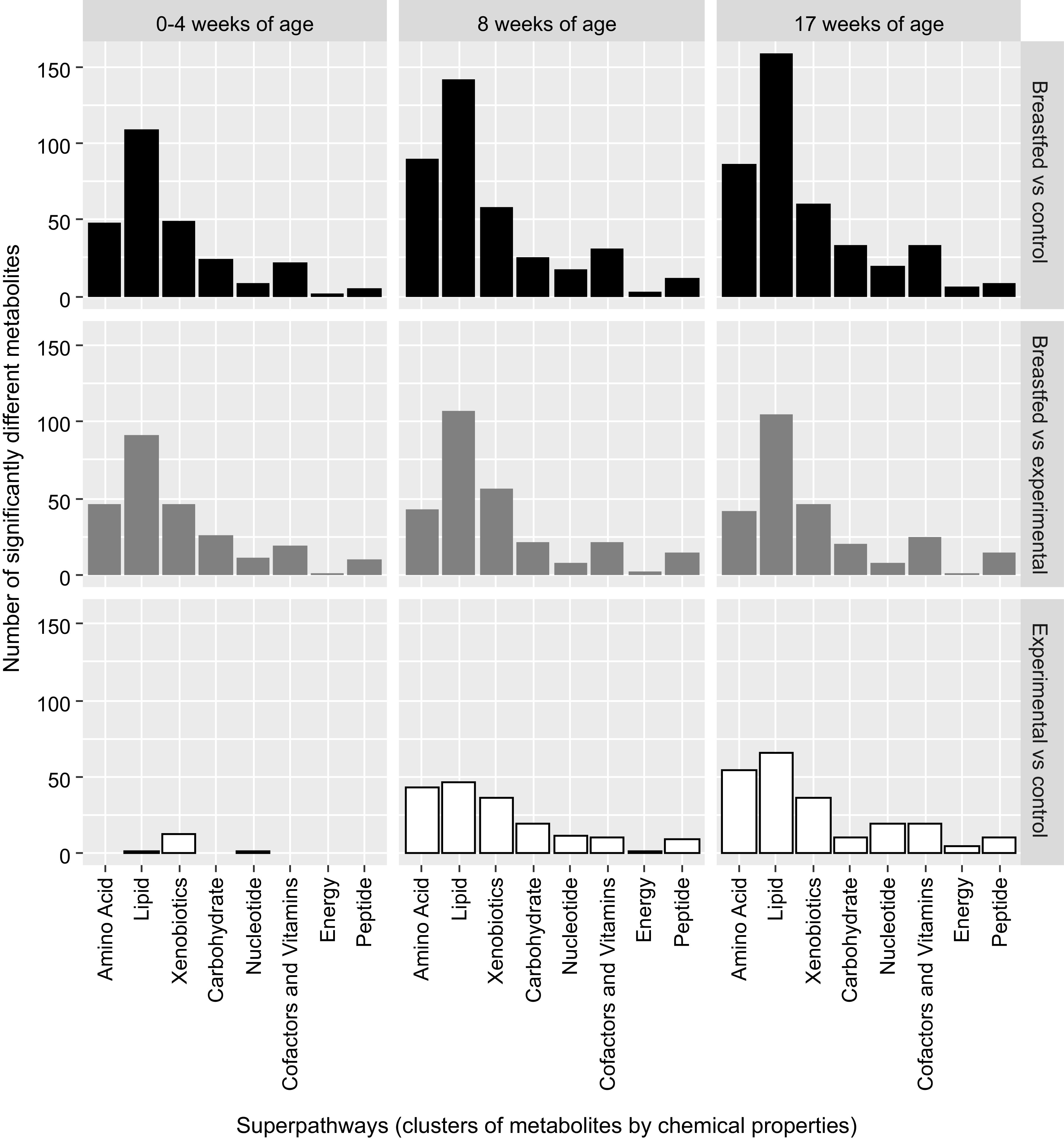
Distribution of the significantly changing metabolites per feeding group per time point, that is, at 0–4 wk of age (baseline), at 8 wk of age, and at 17 wk of age. *Top*: number of different metabolites for the comparisons between the breast-fed reference arm and the control arm. *Middle*: number of different metabolites for the comparisons between the breast-fed reference arm and the experimental arm. *Bottom*: number of different metabolites for the comparisons between the experimental arm and the control arm.

It has been shown in the past decades that the functioning of the colon microbiota largely depends on physiological conditions and the availability of substrates (42–44). The main microbial substrates are host derived, such as mucins (42), or dietary components, such as complex carbohydrate and protein structures that escape host digestion in the upper parts of the gastrointestinal tract (43). The majority of the members of gut microbiota preferentially ferment carbohydrates and only switch to protein fermentation when carbohydrates are depleted (44). These fermentation processes in the gut yield mainly the short-chain fatty acids (SCFAs) acetate, butyrate, and propionate but can also produce formate, valerate, caproate, and branched-chain fatty acids (BCFAs), such as isobutyrate, 2-methylbutyrate, and isovalerate (45). Although SCFAs dominate the metabolite output, the gut microbiota has long been known to produce many more metabolites such as essential amino acids, vitamins, and secondary bile acids (14). The metabolome data presented here allowed us to identify a baseline difference between formula- and human milk-fed infants, which persisted in the first 4 mo of the study. Most of these differential metabolites were amino acids, lipids, xenobiotics, carbohydrates, and vitamins and cofactors (Fig. 5). Interestingly, this baseline difference was extended to nearly twice as many metabolites in the control formula-fed infants at 4 mo of age, whereas in the experimental formula-fed infants, this baseline gap was not further extended. This increase could not be associated with the intake of supplemental foods, as these samples were collected from infants who were exclusively formula-fed throughout the study.

To date, few studies have been reported that use untargeted metabolomics on fecal samples from healthy human milk- and formula-fed infant, and consequently, interpretation of metabolic profiles is challenging. In one of the first reported studies using untargeted fecal metabolomics, four human milk-fed infant stools were compared with four formula-fed infant stools. In this study, Chow and colleagues (25) reported 14 metabolites to be potential markers for human milk feeding. Although this was a relatively small study, we detected 12 out of these 14 markers in our fecal sample set. Similar patterns were shown here between the breast-fed reference arm and both formula-fed arms for the identified human milk oligosaccharides (fucose, 2-fucosyllactose, and lacto-*N*-fucopentaose) and 1-palmitoylglycerophosphocholine. Moreover, phosphate and myo-inositol could be confirmed here as human milk-feeding markers and were, interestingly, also significantly higher in experimental compared with control. l-Lactate (as measured in the targeted physiological measurements) was higher in the breast-fed reference samples when compared with control but even higher in the experimental arm, which could very well be a reflection of the dominance of the *Bifidobacterium* genus in this study arm. Among the suggested human milk-feeding metabolic markers, namely, linoelaidate (18:2n6), taurocholenate sulfate, and uridine, no (significant) differences were detected. Guanine and 3-(4-hydroxyphenyl)lactate were characteristic for the experimental arm and not different between the breast-fed reference and the control arm. Chow and colleagues (25) also reported 41 metabolites to be potential markers for formula feeding; several of these could be confirmed in the data presented here and were even similar between the breast-fed reference and the experimental arm, such as valerate and isovalerate (also confirmed by the targeted physiological analysis, see Supplemental Table S5, Supplemental Table S6, and Supplemental Fig. S1), the secondary bile acid 7-ketolithocholate, and the vitamin B6 compound pyridoxate.

In a larger study, Wang and colleagues (46) applied a fecal metabolomics approach and identified several metabolites as biomarkers for human milk feeding, that is, 15-methylhexadecanoic acid, galactitol, and maltose, as well as several fecal metabolites as biomarkers for formula feeding, that is, β-alanine, dodecanoic acid, glycolic acid, decanoic acid, and tyramine. Our results are in line with these findings by Wang and colleagues. Here, we confirmed β-alanine, tyramine, and dodecanoic acid to be more present in formula-fed infants. Interestingly, for the previously reported formula-feeding marker β-alanine, we observed similar levels in the experimental arm and the human milk-fed reference infants and significantly higher levels in the control infants. Dodecanoic acid (also known as laurate) was lowest in human milk-fed infants, intermediate in the experimental formula-fed infant, and highest in the control-fed infants. Furthermore, levels of the previously reported human milk-feeding marker galactitol were similar in the experimental and human milk-fed reference infants and higher than the levels observed in the control arm infants. Maltose was also detected but only in measurable amounts at baseline in the human milk-fed samples. In contrast to the findings by Wang and colleagues (46) and our findings reported here, Bazanella and colleagues (41) associated dodecanoic acid with human milk-fed infants (at an age of 4 wk), which demonstrates a clear need for additional validations and for establishing an infant gut metabolome reference database to optimize the biological interpretation of such data.

Previous studies in which metabolomics data were combined with microbiota and host organ profiling have already revealed mechanisms involved in specific host-gut microbiota interactions (47). For example, it has been shown that the metabolite trimethylamine *N*-oxide (TMAO), which is produced by the microbiota from dietary phosphatidylcholine and the red meat component l-carnitine (48, 49), ends up in the bloodstream where the TMAO blood plasma levels are associated with cardiovascular disease and atherosclerosis (48–50). This insight has direct clinal relevance for patients with cardiovascular disease and atherosclerosis and may lead to the development of diagnostics and therapies targeting the gut microbiota. Most of these host-microbiota interactions have been studied in a healthy versus compromised (i.e., diseased or other extreme host phenotype) setting. Not much is known about the variability in the healthy functioning hosts and their microbiota, let alone in the developing infant gut. Given the massive impact of nutrition in early life on the microbiota composition and functioning, as presented here by the numerous differences in fecal metabolomics data, it is imperative that we understand the mechanisms by which nutrition stimulates the growth and activity of specific members of the intestinal microbiota. Our data illustrate that the combination of specific prebiotics and postbiotics triggers responses in the intestinal metabolome that bring formula-fed infants closer toward the metabolite profile observed in the breast-fed infants. Further studies are required to translate these dynamics into physiologically relevant responses. We propose that high-resolution phenotypic profiling by untargeted fecal metabolomics provides a powerful approach to further explore gut microbiota interactions with nutrition and early-life health.

## SUPPLEMENTAL DATA

10.6084/m9.figshare.14729682.v1 Supplemental Figure S1 and Tables S1–S7: https://doi.org/10.6084/m9.figshare.14729682.v1.

## GRANTS

This study was supported by Danone-Nutricia Research Grant NTR3455.

## DISCLOSURES

S. Tims, J. Polman, J. Knol, and G. Roeselers are employees of Danone-Nutricia Research that financed the clinical study (NTR3455) from which the clinical samples originated. A. Rodriguez-Herrera, R. Porcel Rubio, A. Muñoz Hoyos, M. Agosti, G. Lista, L. T. Corvaglia, and J. L. Pérez Navero have no conflicts of interests relevant to this article to disclose.

## AUTHOR CONTRIBUTIONS

A.R.-H., J.L.P.N, S.T., R.P.R., A.M.H., M.A., G.L., L.T.C., and J.K. conceived and designed research; S.T. performed experiments; S.T. and J.P. analyzed data; A.R.-H., G.R., S.T., J.P., and J.K. interpreted results of experiments; S.T. and J.P. prepared figures; S.T. drafted manuscript; A.R.-H., G.R., and S.T. edited and revised manuscript; A.R.-H., G.R., J.L.P.N., S.T., J.P., R.P.R., A.M.H., M.A., G.L., L.T.C., and J.K. approved final version of manuscript.
